# Comparative Evaluation and Correlation of Periodontal Status With Inflammatory Markers in Pregnant Women With or Without Chronic Periodontitis: A Clinico-Hematological Study

**DOI:** 10.7759/cureus.55868

**Published:** 2024-03-09

**Authors:** Vaibhava Raaj, Amit Bhardwaj, Prabhat K Singh, Kajal Sinha

**Affiliations:** 1 Department of Periodontology, Faculty of Dental Sciences, Shree Guru Gobind Singh Tricentenary (SGT) University, Gurugram, IND; 2 Department of Periodontology, Buddha Institute of Dental Sciences and Hospital, Patna, IND; 3 Department of Obstetrics and Gynecology, Netaji Subhas Medical College and Hospital, Patna, IND

**Keywords:** periodontal health, blood biomarkers, second trimester, c-reactive protein, lactate dehydrogenase, interleukin-6

## Abstract

Background

The research delves into the intricate relationship between periodontal health and specific blood biomarkers in pregnant women during their second trimester. It specifically focuses on the levels of interleukin-6 (IL-6), lactate dehydrogenase (LDH), and C-reactive protein (CRP) in those suffering from chronic periodontitis compared to healthy controls.

Methodology

A detailed approach was taken involving 60 pregnant women categorized into two groups based on the presence or absence of chronic periodontitis. Out of 60 pregnant women, 30 had chronic periodontitis, while the other 30 served as controls selected from the regular patient population of the college. The study utilized blood sample analysis and advanced statistical tools for data analysis, ensuring precise and reliable results. Levels of IL-6, LDH, and CRP in those suffering from chronic periodontitis compared to healthy controls were checked.

Results

The findings revealed a notable variance in IL-6, LDH, and CRP levels between the two groups. Women with chronic periodontitis exhibited significantly higher levels of these biomarkers. The statistical analysis reinforced the validity of these differences, highlighting their significance.

Conclusions

The study underscored a clear link between higher levels of IL-6, LDH, and CRP and the presence of chronic periodontitis in pregnant women. These biomarkers emerge as potential indicators for early detection and monitoring of periodontal health in this demographic.

## Introduction

In recent decades, untreated caries and severe periodontitis have emerged as leading causes of global oral health problems, surpassing severe tooth loss [1]. Periodontitis and dental caries are two prevalent oral conditions that can lead to tooth loss, with estimates suggesting that up to 90% of the global population may experience periodontal disease at some point in their lives [2]. Periodontitis, characterized by an inflammatory response triggered by the bacterial biofilm in dental plaque, gradually destroys the periodontium, the supporting tissue for teeth. This infectious condition often begins in childhood or adolescence but may not manifest clinically until early adulthood or later in life. It involves both reversible and permanent histological abnormalities. The initial clinical sign of periodontitis is soft tissue inflammation known as gingivitis, followed by changes in hard tissues, including alveolar bone loss and dental cementum diseases [3]. If left untreated, periodontitis can lead to the degeneration of the periodontal ligament, resulting in increased tooth mobility and eventual tooth loss. Periodontal diseases, along with conditions such as diabetes and obesity, are among the most prevalent inflammatory disorders in humans. Recent research highlights their societal impact due to their high prevalence. Only 1% of patients have healthy periodontitis, and more than 16% of people are diagnosed with advanced periodontitis [4,5].

Periodontal disorders involve a bacterial community in the oral cavity triggering an inflammatory response, leading to bleeding upon probing, periodontal attachment loss, bone resorption, and tooth loss [6-11]. These conditions have been associated with systemic illnesses such as heart disease, diabetes, obesity, and metabolic syndrome, suggesting a common low-grade inflammatory mechanism [12]. Evidence indicates that periodontal infections and locally generated cytokines can enter the bloodstream, potentially causing harm in other parts of the body [13]. Several blood cell components, including erythrocyte sedimentation rate (ESR), C-reactive protein (CRP), interleukin-6 (IL-6), fibrinogen, Von Willebrand factor, and thrombocytes, have been studied in connection with periodontitis [14,15]. Periodontal diseases are also characterized by alterations in systemic blood chemistry. Lactate dehydrogenase (LDH), a biomarker associated with increased cell activity, has been linked to periodontal disease. Elevated salivary LDH levels have shown promise as a convenient screening tool for periodontal conditions, potentially aiding in the assessment of systemic inflammatory loads and cardiovascular risk factors [16,17].

CRP, an indicator of the body’s response to acute inflammation, is elevated in periodontal disease, which is characterized by increased levels of inflammatory cytokines and prostaglandins. The systemic nature of periodontal disorders has become increasingly evident, with research exploring how periodontitis can influence changes in peripheral blood composition [18]. Pregnancy introduces significant physiological changes in women’s bodies, including unique gingival changes such as pregnancy gingivitis, characterized by cellular inflammatory infiltration, proliferative inflammation, and non-specific inflammation. Recent studies have explored the potential impact of periodontitis during pregnancy on systemic inflammatory markers such as CRP, IL-6, and tumor necrosis factor-alpha (TNF-α). These markers have been suggested as early indicators of pregnancy-related issues such as preeclampsia, preterm birth, and low birth weight [5]. However, the relationship between periodontal disease, pregnancy, and inflammatory mediators remains a topic of debate [19].

Periodontal inflammation tends to peak during the third trimester of pregnancy, and research suggests that the onset of gingivitis symptoms typically occurs during the second trimester. It is hypothesized that perio-pathogens from the oral cavity can enter the bloodstream and affect the developing fetus, leading to adverse outcomes [20]. Maternal periodontal disease during pregnancy has been associated with negative health outcomes for the offspring, including birth defects, preeclampsia, preterm birth, and long-term risks such as cardiovascular disease, allergies, and asthma. These findings underscore the systemic implications of periodontal disorders [21,22]. Given these considerations, this study aimed to assess and establish links between periodontal health and blood levels of IL-6, LDH, and CRP among second-trimester pregnant women, regardless of their chronic periodontitis status. The study addresses the complex interplay between periodontal health, systemic inflammation, and pregnancy outcomes, highlighting the need for further investigation and early intervention in periodontal disease management, particularly in pregnant women.

## Materials and methods

The study was designed to collect data from pregnant women who visited the outpatient department of the dental college and hospital. The research process began after obtaining ethical committee approval. The ethical approval for the study was given by the ethical committee of Shree Guru Gobind Singh Tricentenary (SGT) University (approval number: SGTU/FDS/24/1/79/2021/552). Ethical considerations included ensuring that there were no significant risks associated with the pathological examination.

The study involved a total of 60 participants, with 30 having chronic periodontitis, while the other 30 serving as controls selected from the regular patient population of the college. Patients who presented themselves to the outpatient department were thoroughly examined, and the study specifically focused on pregnant patients aged between 25 and 35 years. These participants were then divided into the control group and the chronic periodontitis group. The sample size was calculated using the following formula: n = 2(Zα/2​+Zβ​)2 × σ2​/d2, where n is the required sample size per group, Zα/2​ is the Z-value corresponding to the desired significance level α/2, Zβ​ is the Z-value corresponding to the desired power 1−β, σ2 is the estimated population variance, and d is the desired effect size (mean difference between groups divided by the population standard deviation).

Inclusion criteria were pregnant women in their second trimester (approximately 13-26 weeks gestation) aged between 25 and 35 years, good general health status without any systemic diseases or medical complications, willingness to participate in the study, and providing informed consent. Patients needed to be available for regular follow-up visits during the study. They needed to be non-users of antibiotics for more than two weeks in the past three months. Moreover, we included non-smokers, non-tobacco users, non-alcoholics, and non-drug abusers. Included patients were not undergoing hormonal therapy during the study period.

Exclusion criteria included pregnant women in their first or third trimester of pregnancy; any history of systemic diseases or medical complications; recent or ongoing use of antibiotics for more than two weeks within the past three months; smokers, tobacco users, alcoholics, or drug abusers; and undergoing hormonal therapy during the study period. Those unable or unwilling to provide informed consent or participate in the study procedures and were not available for regular follow-up visits during the study period were also excluded.

Patients in their second trimester, aged 25 to 35 years, with or without clinical symptoms of chronic periodontitis, were assessed in the outpatient department. Informed consent was obtained from all participants, both controls and those with chronic periodontitis, after explaining the procedure and objectives of the study. To estimate inflammatory markers, venous blood samples were collected. Aseptic conditions were maintained during the collection of 3 mL of fasting blood, drawn from the most prominent vein, and stored in test tubes with a tourniquet placed 2 mm above the right cubital fossa.

Serum CRP was analyzed using an automated cartridge-based specific protein analyzer (Mispa i3), with kit AGAPPE, following the manufacturer’s instructions. Blood IL-6 levels were measured using a fully automated autoanalyzer (Roche Diagnostics) and the ELECYS IL-6 kit following the manufacturer’s instructions. Serum LDH was evaluated using the Diatek kit with a Riedle Photometer 5010V5+, a semi-automatic, single-beam filter photometer.

Strict sterilization protocols were maintained throughout the study, and new sterile syringes were used for blood sample collection. In the event of any reported allergic reactions, appropriate drugs were administered to the patients. Importantly, participants had the freedom to discontinue and withdraw from the study at any time without needing to provide any clarification. The Oral Hygiene Index Simplified (OHIS), Plaque Index, and Community Periodontal Index (CPI) were determined for both groups and classified as poor, fair, or good.

Collected data were imported into Excel and analyzed statistically. For the statistical analysis of the data, SPSS version 20.0 (IBM Corp., Armonk, NY, USA) was used at a p-value of 0.05. The data were presented by displaying the frequency of categorical variables and providing the mean ± standard deviation for continuous variables. Student’s t-test was performed to analyze continuous variables.

## Results

The objective of this study was to compare the periodontal health and blood levels of IL-6, LDH, and CRP in pregnant women with and without chronic periodontitis. The study involved 60 female participants aged 25 to 35 years divided into two groups, namely, a control group (without periodontitis) comprising 30 individuals, and a study group (with periodontitis) comprising 30 individuals. Table 1 presents the age distribution of both groups. In the control group (group 1), participants’ ages ranged from 26 to 32 years, with a mean age of 28.00 ± 1.58 years. In the study group (group 2), participants’ ages ranged from 25 to 36 years, with a mean age of 27.57 ± 2.24 years.

**Table 1:** Comparison of mean age of study participants in both groups. P-values <0.05 are considered significant.

Variables	N	Minimum	Maximum	Mean	Standard deviation
Group 1 (without periodontitis)	30	26.00	32.00	28.0000	1.57568
Group 2 (with periodontitis)	30	25.00	36.00	27.5667	2.23889
t-test	1.289				
P-value	0.065				

Despite the slight discrepancy in mean age between group 1 and group 2, a one-sample t-test revealed no statistically significant difference (p > 0.05) in age between the two groups. This suggests that age-related factors did not significantly impact the outcome measures of the study. Hematological parameters, including IL-6, CRP, and LDH, were evaluated in terms of IL-6 levels (pg/mL). Table 2 provides a summary of IL-6 levels in both groups. The findings indicate that participants in group 2, diagnosed with periodontitis, displayed markedly higher mean IL-6 levels compared to those in the control group (group 1). Specifically, the mean IL-6 level in group 2 was 7.87 ± 0.349 pg/mL, notably higher than the mean level of 3.71 ± 0.464 pg/mL observed in group 1. A statistical analysis employing a one-sample t-test confirmed a significant and meaningful difference (p < 0.05) between the two groups regarding IL-6 levels (Table 2).

**Table 2:** Comparison of mean interleukin-6 of study participants in both groups. P-values <0.05 are considered significant.

Variables	N	Minimum	Maximum	Mean	Standard deviation
Group 1 (without periodontitis)	30	3.01	4.55	3.7120	0.46489
Group 2 (with periodontitis)	30	7.14	8.55	7.8693	0.34907
t-test	1.330				
P-value	0.005				

Table 3 demonstrates that participants in group 2, diagnosed with periodontitis, exhibited significantly higher mean LDH (µ/L) levels compared to those in the control group (group 1). Specifically, the mean LDH level in group 2 was 522.649 ± 31.158 µ/L, notably higher than the mean level of 348.7 ± 49.51 µ/L observed in group 1. Statistical analysis using a one-sample t-test confirmed a significant difference (p < 0.05) between the two groups regarding LDH levels. The analysis of LDH levels in both groups is summarized in Table 3.

**Table 3:** Comparison of mean lactate dehydrogenase of study participants in both groups. P-values <0.05 are considered significant.

Variables	N	Minimum	Maximum	Mean	Standard deviation
Group 1 (without periodontitis)	30	236.30	414.50	348.7000	49.51444
Group 2 (with periodontitis)	30	471.70	574.80	522.6497	31.15819
t-test	1.554				
P-value	0.012				

The data concerning CRP (mg/L) levels in both study groups are presented in Table 4. As depicted in Table 4, the mean CRP level in group 2 was recorded at 7.653 ± 0.882 mg/L, while in group 1, it was significantly lower at 2.27 ± 1.483 mg/L. A statistically significant difference (p < 0.05) between the two groups concerning CRP levels was confirmed using a one-sample t-test.

**Table 4:** Comparison of mean C-reactive protein of study participants in both groups. P-values <0.05 are considered significant.

Variables	N	Minimum	Maximum	Mean	Standard deviation
Group 1 (without periodontitis)	30	0.58	8.09	2.2743	1.48276
Group 2 (with periodontitis)	30	4.62	8.91	7.6530	0.88250
t-test	1.461				
P-value	0.034				

The OHIS index, categorized as poor, fair, and good, was utilized to classify all participants in both study groups. This classification was then employed to assess the correlation between periodontal health, as determined by OHIS, and the levels of IL-6, CRP, and LDH. Table 5 demonstrates that in group 1, the mean levels of all hematological parameters were notably higher in individuals with a fair OHIS index compared to those with a good OHIS index.

**Table 5:** Association of hematological parameters with OHIS index in group 1. P-values <0.05 are considered significant. SD: standard deviation; IL-6: interleukin-6; LDH: lactate dehydrogenase; CRP: C-reactive protein; OHIS: Oral Hygiene Index Simplified

OHIS	IL-6 (pg/mL)		LDH (µ/L)		CRP (mg/L)	
	Mean	SD	Mean	SD	Mean	SD
Poor	-	-	-	-	-	-
Fair	3.8993	0.36759	367.0200	46.44419	2.8747	1.70787
Good	3.5247	0.48719	330.3800	46.92787	1.6740	0.93009
t-test	2.819		28.150		1.029	
P-value	0.054		0.044		0.031	

When assessing the correlation between the status of periodontal health using the OHIS index and the mean hematological parameters, a one-sample t-test revealed a significant and statistically meaningful difference (p < 0.05) between OHIS index grades and IL-6, LDH, and CRP levels in group 1.

In group 2, all patients had a poor OHIS index, indicating that their periodontal health was compromised (Table 6).

**Table 6:** Association of hematological parameters with OHIS index in group 2. SD: standard deviation; IL-6: interleukin-6; LDH: lactate dehydrogenase; CRP: C-reactive protein; OHIS: Oral Hygiene Index Simplified

OHIS	IL-6 (pg/mL)		LDH (µ/L)		CRP (mg/L)	
	Mean	SD	Mean	SD	Mean	SD
Poor	7.8693	0.34907	522.6497	31.15819	7.6530	0.88250
Fair	-	-	-	-	-	-
Good	-	-	-	-	-	-

These findings underscore the substantial impact of periodontal health on hematological parameters, as reflected in the higher mean levels of all hematological parameters in group 2 compared to group 1. Group 2 patients, characterized by poorer periodontal health, exhibited elevated levels of IL-6, LDH, and CRP. To explore the association of hematological parameters with the Plaque Index, individuals in both groups were categorized based on the Plaque Index as poor, fair, and good.

As shown in Table 7, in group 1, the mean levels of all hematological parameters were significantly higher in cases with a fair Plaque Index compared to those with a good Plaque Index.

**Table 7:** Association of hematological parameters with Plaque Index in group 1. P-values <0.05 are considered significant. SD: standard deviation; IL-6: interleukin-6; LDH: lactate dehydrogenase; CRP: C-reactive protein

Plaque Index	IL-6 (pg/mL)		LDH (µ/L)		CRP (mg/L)	
	Mean	SD	Mean	SD	Mean	SD
Poor	-	-	-	-	-	-
Fair	3.8636	0.42517	373.5818	42.77129	2.8745	1.96857
Good	3.6242	0.47495	334.2947	48.33448	1.9268	1.02058
t-test	1.718		22.718		1.665	
P-value	0.042		0.0118		0.028	

Evaluating the correlation between periodontal health, as determined by the Plaque Index, and hematological parameters, a one-sample t-test revealed a significant statistical difference (p < 0.05) between the Plaque Index and IL-6, LDH, and CRP levels in group 1. The Plaque Index was poor for all patients in group 2, emphasizing that the mean levels of all hematological parameters were higher in group 2 compared to group 1 (Table 8).

**Table 8:** Association of hematological parameters with Plaque Index in group 2. SD: standard deviation; IL-6: interleukin-6; LDH: lactate dehydrogenase; CRP: C-reactive protein

Plaque Index	IL-6 (pg/mL)		LDH (µ/L)		CRP (mg/L)	
	Mean	SD	Mean	SD	Mean	SD
Poor	7.8693	0.34907	522.6497	31.15819	7.6530	0.88250
Fair	--	-	-	-	-	-
Good	-	-	-	-	-	-

This observation underscores the significant influence of periodontal health on hematological parameters. Patients in group 2 exhibited poor periodontal health, leading to elevated levels of IL-6, LDH, and CRP.

Table 9 illustrates that the mean levels of all hematological parameters were significantly higher in cases with a code 0 or 1 in the CPI compared to those with a code 2 or 3 in group 1.

**Table 9:** Association of hematological parameters with CPI in group 1. P-values <0.05 are considered significant. SD: standard deviation; IL-6: interleukin-6; LDH: lactate dehydrogenase; CRP: C-reactive protein; CPI: Community Periodontal Index

CPI	IL-6 (pg/mL)		LDH (µ/L)		CRP (mg/L)	
	Mean	SD	Mean	SD	Mean	SD
Poor	-	-	-	-	-	-
Fair	3.6410	0.49975	339.6050	52.17892	2.1140	1.71392
Good	3.8540	0.36815	366.8900	40.00751	2.5950	0.84346
t-test	1.189		2.189		1.718	
P-value	0.018		0.051		0.033	

Analyzing the relationship between periodontal health status assessed by the CPI and mean hematological parameters, a one-sample t-test revealed a significant statistical difference (p < 0.05) between the CPI and IL-6, LDH, and CRP levels in group 1. All patients in group 2 had CPI code 4, demonstrating higher mean levels of all hematological parameters in group 2 compared to group 1 (Table 10).

**Table 10:** Association of hematological parameters with CPI in group 2. SD: standard deviation; IL-6: interleukin-6; LDH: lactate dehydrogenase; CRP: C-reactive protein; CPI: Community Periodontal Index

CPI	IL-6 (pg/mL)		LDH (µ/L)		CRP (mg/L)	
	Mean	SD	Mean	SD	Mean	SD
Poor	7.8693	0.34907	522.6497	31.15819	7.6530	0.88250
Fair	-	-	-	-	-	-
Good	-	-	-	-	-	-

This revealed that periodontal health has a significant impact on all the hematological parameters. Group 2 patients had pathological pockets of more than 4 mm, thus revealing higher levels of IL-6, LDH, and CRP.

## Discussion

Periodontitis is a complex chronic infection that affects the periodontium, the supporting framework of teeth. It often remains silent in infancy or youth but becomes clinically evident in early adulthood or even later. This condition encompasses a wide range of symptoms, progressing from gingivitis to alveolar bone loss and dental cementum diseases. Left untreated, periodontitis can lead to tooth mobility and eventual loss [2]. Periodontal diseases are not confined to oral health; they have systemic implications driven by a low-grade inflammatory load. Extensive research has explored the connections between periodontitis and various blood cell components, including ESR, CRP, IL-6, fibrinogen, Von Willebrand factor, and thrombocytes [23]. Cytokines such as IL-1β, TNF-α, IL-6, and RANKL are pivotal in managing the immune response in periodontal diseases. Additionally, several cell activity indicators, such as alkaline phosphatase, LDH, aspartate aminotransferase, alanine aminotransferase, and matrix metalloproteases, have been linked to periodontal health. Notably, there is also emerging evidence of associations between periodontitis and Parkinson’s disease [24].

Pregnancy can lead to various gingival changes, including pregnancy gingivitis characterized by cellular infiltration, non-specific inflammation, and proliferative and vascular inflammation. Recent studies have suggested potential links between periodontitis during pregnancy and systemic inflammatory markers such as CRP, IL-6, and TNF-α. These markers may serve as early indicators of pregnancy complications such as preeclampsia, preterm birth, and low birth weight. However, the relationship between periodontal disease, pregnancy, and the role of inflammatory mediators remains a subject of debate [25-28]. This study aimed to investigate the correlation between periodontal health and the blood levels of IL-6, LDH, and CRP in pregnant women during their second trimester, irrespective of their periodontal status.

The study involved 60 female patients aged 25-35 years, with 30 having chronic periodontitis and 30 controls. The study sought to compare the periodontal condition of these two groups with their blood levels of IL-6, LDH, and CRP as outcome markers. Ensuring no statistically significant age differences between the groups was essential to eliminate potential confounding factors [26]. The study focused on the analysis of IL-6, LDH, and CRP levels. Results indicated that patients with periodontitis had significantly higher levels of IL-6, LDH, and CRP compared to the control group, highlighting an association between elevated biomarker levels and periodontitis. This finding aligns with previous research reporting increased biomarker levels in individuals with periodontitis [29].

The research also assessed periodontal health using OHIS, Plaque Index, and CPI, classifying them as poor, fair, or good. The study revealed that periodontal health was significantly correlated with IL-6, LDH, and CRP levels. Patients with poor periodontal health exhibited higher levels of these hematological parameters, underscoring the impact of periodontal health on biomarker levels. The findings of this study indicate that pregnant women with compromised periodontal health exhibit elevated levels of IL-6, LDH, and CRP. These hematological biomarkers offer clinical benefits when assessed alongside conventional investigations. They can assist in evaluating disease severity, predicting its activity, and assessing future disease progression. Consequently, these parameters can help determine immediate and future treatment needs [30,31].

The study has some limitations such as its relatively small sample size. Future research should involve larger longitudinal studies for more robust results. Additionally, further studies should explore a broader range of biomarkers, both in serum and saliva, to assess periodontal disease. Establishing reference values for biomarkers to distinguish periodontitis from normal periodontal health in pregnant women should also be a focus of prospective studies and clinical trials.

## Conclusions

Patients with poor periodontal health had elevated levels of IL-6, CRP, and LDH biomarkers. These findings indicate the potential of hematological biomarkers to assess disease severity, predict activity, and evaluate progression. Pregnant females with periodontal issues had significantly raised IL-6, LDH, and CRP levels, emphasizing their clinical relevance alongside conventional investigations. Future research with larger samples and additional biomarkers is recommended for early periodontitis diagnosis in pregnant women. The study concludes that strong correlations exist between CRP, LDH, IL-6 levels, periodontitis, and conventional indices in pregnant females, emphasizing the need for early diagnosis.
